# Haploinsuficiencia de la proteína PUR-a por la mutación *de novo* de cambio de sentido c.692T>C (p-Phe231Ser) del gen *PURA:* primer reporte en Colombia

**DOI:** 10.7705/biomedica.7286

**Published:** 2024-11-06

**Authors:** Sandra Milena Cerón, Daniel Alejandro Pérez, Julio Herberth Montaño, María Amparo Acosta

**Affiliations:** 1 Facultad de Ciencias de la Salud, Universidad del Cauca, Popayán, Colombia Universidad del Cauca Facultad de Ciencias de la Salud Universidad del Cauca Popayán Colombia; 2 Departamento de Pediatría, Facultad de Ciencias de la Salud, Universidad del Cauca, Popayán, Colombia Universidad del Cauca Departamento de Pediatría Facultad de Ciencias de la Salud Universidad del Cauca Popayán Colombia; 3 Hospital Universitario San José, Popayán, Cauca, Colombia Hospital Universitario San José Popayán Cauca Colombia

**Keywords:** enfermedades del sistema nervioso, trastornos del neurodesarrollo, niño, Nervous system diseases, neurodevelopmental disorders, child

## Abstract

Se presenta el primer caso documentado en Colombia del síndrome PURA, una enfermedad neurológica rara. Esta afección resulta de mutaciones del gen *PURA,* localizado en el cromosoma 5, que producen haploinsuficiencia de la proteína PUR-a. Esta proteína es esencial para el desarrollo temprano del cerebro y la función neuronal. El paciente, un niño de siete años, comenzó a presentar movimientos distónicos de las manos a los 14 días de vida. A los seis años, se le diagnosticó retraso en el neurodesarrollo, hipotonía generalizada, episodios frecuentes de apnea y dificultad para la deglución. Aunque inicialmente se consideraron otras condiciones, como la distrofia muscular de Duchenne y la lipofuscinosis ceroidea neuronal, la secuenciación completa del exoma reveló la variante patógena c.692T>C (p.Phe231Ser) en el exón 1 del gen *PURA,* no registrada previamente en otros pacientes. Este hallazgo permitió un enfoque de manejo integral, con el cual se abordaron las características y alteraciones clínicas del paciente. Dado que el síndrome PURA no figura en la lista de enfermedades huérfanas o raras reconocidas por el Ministerio de Salud y Protección Social de Colombia, este reporte podría influir en su reconocimiento oficial.

El caso demuestra la importancia de considerar diagnósticos raros en pacientes con síntomas neurológicos poco comunes y subraya la utilidad de la secuenciación genómica para el diagnóstico. Además, enfatiza la necesidad de colaboración en el área de la salud para optimizar el cuidado de los pacientes con síndrome PURA y otras enfermedades semejantes.

## Abstract (en)

We present the first documented case of PURA syndrome in Colombia. This rare neurological disease results from mutations in the *PURA* gene located on chromosome 5, leading to haploinsufficiency of the PUR-a protein. This protein is essential for early brain development and neuronal function.

The patient, a seven-years-old boy, started showing dystonic hand movements at 14 days of age; at six, he had neurodevelopmental delay, generalized hypotonia, frequent episodes of apnea, and swallowing difficulties.

Although other conditions were initially considered, such as Duchenne muscular dystrophy and neuronal ceroid lipofuscinosis, a whole exome sequencing revealed the pathogenic variant c.692T>C (p.Phe231Ser) in the exon 1 of the *PURA* gene, not previously reported in other patients. With this finding, we adopted a comprehensive management approach addressing the patient's characteristics and alterations.

Since the PURA syndrome is not on the list of orphan/rare diseases recognized by the Colombian *Ministerio de Salud y Protección Social,* we hope our report will contribute to its official recognition.

The case shows the importance of considering rare diagnoses in patients with uncommon neurological symptoms, underlining the usefulness of genomic sequencing in diagnosis and the need for collaboration to optimize healthcare for patients with PURA syndrome and similar diseases.

Existe una gran cantidad de enfermedades denominadas huérfanas o raras, con muy baja prevalencia y tan pocos casos reportados, que su diagnóstico, seguimiento y tratamiento son complejos. En este grupo de enfermedades se encuentra el síndrome PURA (OMIM_616158), una afección de origen genético, con herencia autosómica dominante y alteraciones de predominio neurológico. Recibe su nombre por la alteración de la proteína de unión a elementos ricos en purina a en las personas que la padecen. Esta proteína es codificada por el gen que recibe el mismo nombre, *PURA,* ubicado en el brazo largo del cromosoma 5 a nivel de q31.2-q31.3 ^1^. Dicho síndrome se puede presentar con limitación leve a moderada en el desarrollo intelectual, convulsiones, hipotonía, movimientos anormales, dificultad para respirar, deglutir o controlar la temperatura corporal, sueño excesivo y alteraciones cardiacas, gastrointestinales y visuales, entre otras ^2^^,^^3^. La función normal de dicha proteína está relacionada con el crecimiento y la división de las células nerviosas, la formación y la maduración de la mielina, y la replicación del ADN. Sus alteraciones pueden deberse a mutaciones puntuales, deleciones o inserciones en el gen ^4^.

En Colombia, el Ministerio de Salud y Protección Social publicó la última actualización del listado de enfermedades huérfanas en el 2023. A pesar de que el síndrome PURA está claramente clasificado como enfermedad huérfana en otros países, en Colombia aún no se cataloga como tal ^5^. Además, el Instituto Nacional de Salud en su informe anual del 2022 sobre enfermedades huérfanas-raras ^6^, no registró información respecto a esta condición, por lo que este se trataría del primer caso reportado en el país.

## Descripción del caso

Se trata de un paciente de siete años y sexo masculino, natural y procedente de Popayán (Cauca). Los padres negaron consanguinidad y exposición a teratógenos. El padre era un hombre sano de 32 años y, la madre, una mujer sana de 23 años, con antecedente de dos gestaciones y dos partos; su primera hija nació sana.

El paciente fue producto del segundo embarazo, no planeado, sin historial de atención anterior a la concepción. Recibió atención prenatal temprana desde la cuarta semana, seis controles en total; su ingestión de micronutrientes fue adecuada. Se le practicaron cuatro ecografías obstétricas, sin hallazgos anormales.

El paciente nació a las 38 semanas de gestación por parto vaginal atendido en una institución de salud; su peso al nacer fue de 3.340 g, su talla fue de 51 cm y el puntaje de Apgar fue adecuado. Presentó ictericia neonatal no hemolítica con duración de 45 días, con valores de bilirrubinas entre 5,4 y 12,8 mg/dl, por lo cual recibió fototerapia. Sin embargo, presentó hipoglucemia neonatal, cianosis e hipotonía; y, además, dificultad respiratoria por neumonía y sepsis neonatal temprana.

Por otra parte, a los 14 días de vida presentó movimientos distónicos en ambas manos, de cuatro a cinco veces al día, sin pérdida del conocimiento, que se diagnosticaron como episodios convulsivos. Al inicio de la lactancia, se observaron episodios graves y frecuentes de apnea del sueño; también, hubo persistencia de la hipotonía generalizada, varios episodios de neumonía por aspiración y trastornos de la deglución.

### Hallazgos clínicos

Durante el examen físico a los 6 años de edad, las medidas antropométricas fueron: peso, 21 kg; talla, 116 cm; relación talla/edad, entre 0 y -1 DE (desviación estándar); relación IMC/edad, 0 + 1 DE; envergadura, 108 cm; puntaje esperado menos total (E-T) = -8; segmento pubis-cabeza, 58 cm; segmento pubis-pie, 59 cm; SS/SI, 0,98; y perímetro cefálico, 52 cm.

**Figura 1:**
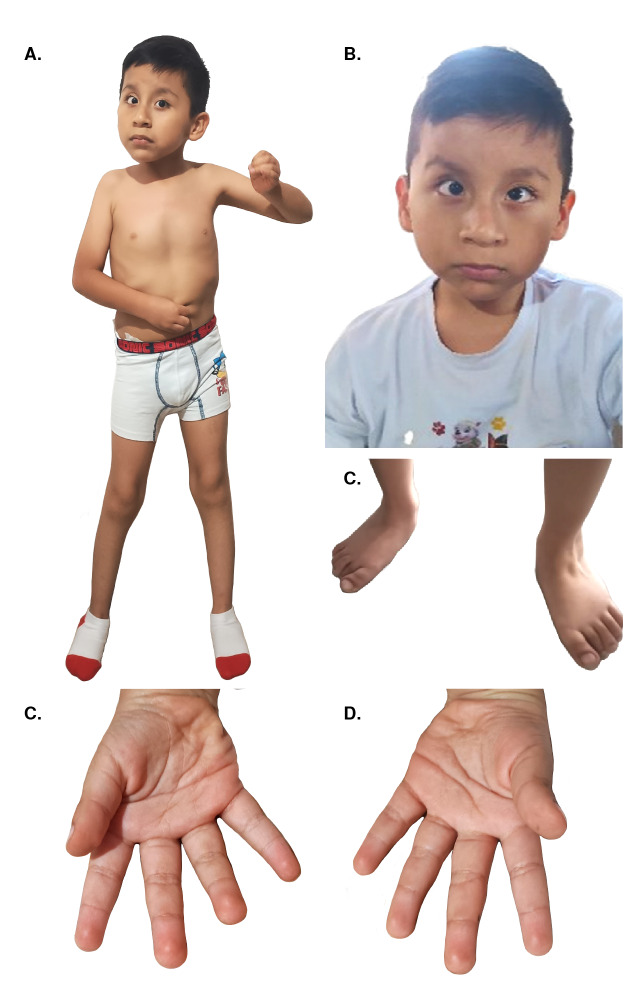
A) Fenotipo general, vista frontal. B) Hipertelorismo, endoforia bilateral y, orejas de implantación baja. C) Pie plano. D) Pliegue único de flexión en la mano derecha. E) Mano izquierda normal

Como características morfológicas, se observó: braquicefalia, plagiocefalia occipital derecha, pabellones auriculares de implantación baja, cara ovalada, endoforia bilateral, pseudoestrabismo, epicanto bilateral, hipertelorismo ocular, distancia intercántica interna de 40 mm, distancia intercántica externa de 140 mm, pupilas isocóricas fotorreactivas, nariz sin desviación del tabique nasal, puente nasal normal, filtro o surco nasolabial de 20 mm, lengua y úvula sin alteraciones, paladar alto, diastemas dentales y esmalte dental de regular calidad (figura 1). Además, presentaba: *pectus excavatum,* abdomen normal y escoliosis lumbar derecha; prepucio redundante y testículos descendidos; extremidades móviles, simétricas e hipotónicas; mano derecha con pliegue único de flexión, y pie plano bilateral.

En el examen neurológico se encontró un paciente alerta, con afasia motora (emitía sonidos incomprensibles); no tenía señales de trastornos auditivos y la mayoría de los pares craneales eran normales, a excepción del VI par craneal bilateral. Además, había pseudoestrabismo convergente e hipotonía de predominio axial, con reflejos presentes (++); requería ayuda para la marcha, fuerza de 3/5 en todas las extremidades, sensibilidad conservada y falta de control de esfínteres.

En el cuadro 1 se presenta el enfoque utilizado para abordar el caso, destacando los posibles diagnósticos diferenciales.

**Cuadro 1:** Diagnósticos diferenciales que permiten enfocar el caso clínico.

Diagnósticos diferenciales	Presencia	Estudio	Fecha	Resultado e interpretación
Distrofia muscular de	No	- Detección de deleciones y duplicaciones del gen de la distrofina por la técnica de amplificación por ligación de sondas múltiples	4 de agosto de 2015	Sin evidencia de variantes patogénicas
Duchenne/Becker				

		- Secuenciación del gen de la distrofia muscular de Duchenne		
Disfagia	Sí	Videofluoroscopia de deglución	21 de octubre de 2016	Disfagia orofaríngea, microaspiraciones y trazos de aspiración durante la deglución de líquidos claros, por ascenso laríngeo disminuido, sin respuesta de tos
Reflujo gastroesofágico de grado I	Sí	Radiografía de vías digestivas altas con tránsito intestinal	11 de noviembre de 2016	Algunos pequeños eventos de reflujo gastroesofágico de grado I y marco duodenal de posición normal
Enfermedad de Niemann-Pick A/B	No	Determinación estructural de lisosfingomielina-509 por cromatografía líquida de alta resolución y espectrometría de masas	8 de marzo de 2017	Resultado: 0,4 ng/ml Poca probabilidad de padecer enfermedad de Niemann-Pick
Trastorno deglutorio (disfagia)	Sí	Faringografía y esofagograma con cine o video (estudio de la deglución)	15 de septiembre de 2017	- Disfagia orofaríngea caracterizada por déficit en los procesos de masticación
				- Penetraciones al vestíbulo laríngeo con líquidos espesos (presentación menor del 10 % de las degluciones realizadas con esta consistencia) y microaspiraciones durante la deglución con líquidos claros por ascenso laríngeo disminuido, sin reacción de protección
Distrofia muscular avanzada y fibrosis	Sí	Biopsia muscular con tinciones de rutina	15 de diciembre de 2017	- Músculo estriado con variación en el tamaño de las miofibras sin internalización de los núcleos
				- Áreas de fibrosis en el endomisio en las que hay algunas fibras necróticas
				- Hay varias áreas donde las fibras pierden sus estrías y se homogenizan formando fibrosis.
Síndrome de apnea obstructiva del sueño severo	Sí	Polisomnografías	26 de septiembre de 2017	- Síndrome grave de hipopnea
				- Deficiencia de sueño del 94 %
				- Neumología recomienda a los padres el uso continuo de presión positiva continua en las vías respiratorias durante el sueño
			29 de junio de 2018	
Lipofuscinosis ceroidea neuronal infantil (CLN1),	No	Medición de:Palmitoil tioesterasa (CLN1) y tripeptidil peptidasa (CLN2)	2 de febrero de 2018	Resultado: CLN1: 11 mmol/hora/ml (valores de referencia: 5,6-15)
Lipofuscinosis ceroidea neuronal tipo 2 (CLN2)	Sí	Tomografía computada de tórax	14 de marzo de 2018	CLN2: 6,1 mmol/hora/ml (valores de referencia: 4,0-23)
Microinspiraciones y episodios de neumonía a repetición				El parénquima pulmonar presenta opacidades en vidrio esmerilado, parcheadas, peribroncovasculares distales y atelectasias subsegmentarias en el segmento anterior del lóbulo superior izquierdo y el segmento posterior del lóbulo superior derecho. Se observan atelectasias gravitacionales bilaterales.
Neuropatía	No	Electromiografía de miembros superiores e inferiores	9 de marzo de 2018	Estudio normal, no hay signos electrofisiológicos de neuropatía.
Alteraciones estructurales o funcionales del corazón	No	Ecocardiograma	21 de mayo de 2019	- No se observa conducto arterioso persistente.
				- Presenta patrón coronario, pericardio y función ventricular normales.
				- No hay signos de hipertensión pulmonar.
Aneuploidías en células individuales	No	Hibridación genómica comparativa basada en microarreglos (aCGH) 180 K, posnatal, a partir de muestra sangre periférica	15 de noviembre de 2019	Sin variaciones en el número de copias relacionadas con la enfermedad
Síndrome PURA por mutación de *novo*	Sí	Exoma trío	30 de junio de 2020	Síndrome PURA: c.692T>C (p.Phe231Ser), variante patogénica, de herencia autosómica dominante
Alteraciones estructurales y/o funcionales del riñón	No	Ecografía renal y de vías urinarias	15 de febrero de 2021	- Riñones de forma y volumen normales, con ecogenicidad homogénea
				- No hay evidencia de dilatación del sistema pielocalicial pélvico ureteral
				- La relación corticomedular se encuentra conservada.
				- Vejiga sin alteraciones
Epilepsia	Sí	Informe de electroencefalografía (telemetría para epilepsia)	22 de abril de 2021	Actividad epileptogénica interictal temporal bilateral
Enfermedad de Pompe	No	Actividad enzimática de la α-glucosidasa		Normal

### Diagnóstico molecular

A la edad de cinco años se hizo la secuenciación completa del exoma y se identificó una variante heterocigota, probablemente patógena, en el exón 1 del gen *PURA:* c.692T>C (p.Phe231Ser).

### Consideraciones éticas

Mediante firma del consentimiento informado, el padre autorizó la revisión de la historia clínica, la toma de registros audiovisuales, y la realización y publicación del reporte de caso.

## Discusión

El síndrome PURA es una enfermedad neurológica de origen genético, producto de la mutación del gen *PURA,* ubicado en el cromosoma 5, locus q31.2-q31.3, cuya secuencia codifica para una proteína activadora de la transcripción: la proteína a de unión a elementos ricos en purinas ^7^. Esta proteína participa en el crecimiento y la división neuronal, la maduración de las dendritas y la transmisión de sinapsis ^8^.

**Figura 2:**
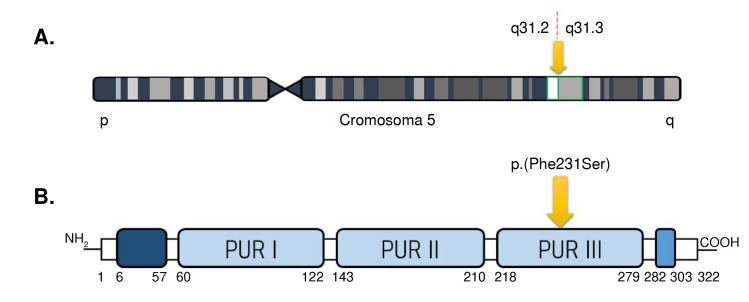
A) Localización del gen *PURA* en el cromosoma 5, locus q31.2-q31.3. B) Proteína Pur-a y ubicación de la variante patógena del paciente.

Los primeros reportes del síndrome PURA aparecieron en la literatura médica en el año 2011, con el nombre de síndrome de microdeleción en 5q31.3. Esta enfermedad está clasificada dentro del grupo de las raras o huérfanas debido a su escasa prevalencia, ya que en el mundo afecta aproximadamente a 500 personas entre adultos y niños ^9^^,^^10^. Los defectos asociados con este síndrome son causados por mutaciones del gen del exón único *PURA* que, como se mencionó, codifica para PUR-α, una proteína multifuncional muy conservada y expresada de manera ubicua, conformada por 322 aminoácidos. Estructuralmente, tiene un dominio N-terminal rico en glicina (6-57 aminoácidos), tres repeticiones centrales conservadas Pur I-II-III (60-279 aa) y un dominio C-terminal rico en glutamina y glutamato (282-303aa) (figura 2) ^11^.

La proteína pur a es miembro de la familia proteica PUR y, al actuar como factor de transcripción, puede combinarse directa e indirectamente con el ADN, promoviendo o inhibiendo la transcripción génica. Esta transcripción es esencial para llevar a cabo los procesos relacionados con el desarrollo posnatal normal de las células de la médula ósea, los músculos y el cerebro, ya que la PURA participa en la proliferación neuronal, la maduración de las dendritas, el transporte de ARNm a los sitios de traducción en las neuronas del hipocampo, y la formación y maduración de la mielina. Por lo tanto, las mutaciones relacionadas con el gen *PURA* son responsables de retrasos moderados a graves del neurodesarrollo ^12^^-^^14^.

**Figura 3:**
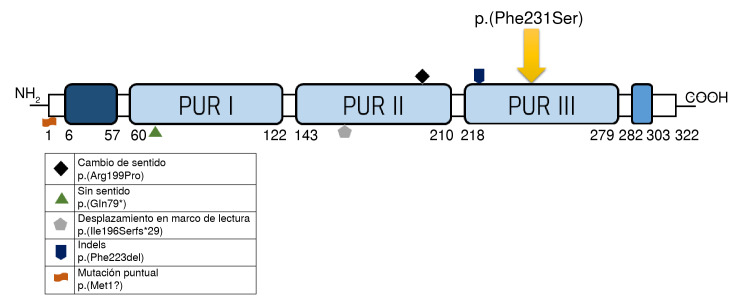
Ubicación y ejemplos de variantes patogénicas *de novo*.

Los trastornos del desarrollo neurológico relacionados con alteraciones en el gen *PURA* incluyen el síndrome PURA, causado por una variante de secuencia patogénica heterocigota del gen en 90 % de los casos, y el síndrome de deleción 5q31.3, causado por una deleción del locus 5q31.3 que abarca todo o parte del gen *PURA* en 10 % de los casos. A la fecha, se conocen 61 variantes diferentes *de novo* de la secuencia intragénica del gen *PURA,* que incluyen mutaciones puntuales, cambio de sentido, sin sentido, desplazamiento del marco de lectura e inserciones o deleciones (indels) (figura 3). Estas variantes llevan a la haploinsuficiencia de PUR-a y a las manifestaciones clínicas descritas ^15^.

El síndrome PURA como tal es un trastorno monogénico esporádico y se identificó por primera vez mediante la secuenciación completa del exoma, que permitió el diagnóstico de variantes patogénicas heterocigotas *de novo* en 15 pacientes en el 2014 ^16^.

**Figura 4:**
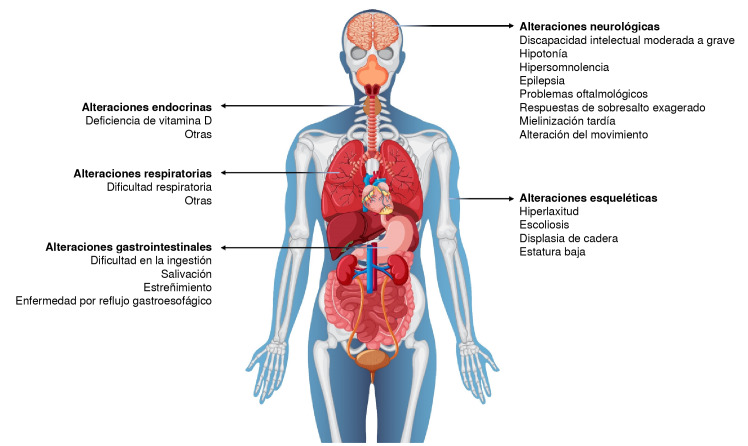
Manifestaciones clínicas del síndrome PURA [Body vectors by Vecteezy (https://www.vecteezy.com/free-vector/body)]

Las características de los individuos con síndrome de deleción 5q31.3 se superponen con las de aquellos con una variante patogénica en el gen *PURA* e incluyen: hipotonía neonatal, trastornos de la deglución y respiratorios, así como discapacidad intelectual grave y episodios convulsivos ^7^^,^^17^^,^^18^ (figura 4).

Cabe destacar que los individuos con deleciones que también incluyen el gen contiguo *NRG2* o más grandes (que abarcan múltiples genes además de *PURA* y *NRG2),* presentan un fenotipo más grave con distintos dimorfismos faciales que los individuos con una variante patógena intragénica ^18^.

Mediante el análisis de secuenciación de última generación, se identificó en este caso una variante heterocigota de tipo cambio de sentido, que ocurrió *de novo* y que consiste en la sustitución de la base nitrogenada timina por citosina en la posición 692, lo cual genera un cambio del aminoácido fenilalanina por serina en la posición 231 de la secuencia proteica de PUR-a (cuadro 2), una posición muy conservada a nivel evolutivo.

**Cuadro 2:** Reporte de hallazgos obtenidos mediante secuenciación masiva y Sanger a partir del ADN del paciente y sus progenitores

Gen	Mutación	Id SNV	Clasificación de variante	Cigosidad			Enfermedad asociada	Tipo de herencia
				Paciente	Padre	Madre		
*PURA* (NM_005859.5	c.692T>C (p.Phe231Ser)	rs1554129113	Probablemente patogénica	Heterocigoto	No presente	No presente	Síndrome PURA (OMIM_616158)	Autosómica dominante

Fuente: SYNLAB, exámenes y valoraciones, 13 de julio de 2020, conservado en el Servicio de Pediatría del Hospital Universitario San José, Popayán, Colombia

Id SNV: identificación de variante de nucleótido único

Esta variante aparece descrita como patógena en la base de datos de variantes ClinVar, pero no se menciona en otras bases de datos ni en la literatura científica consultada hasta la fecha. Se sugiere la aparición *de novo* de esta variante, probablemente patógena, ya que en el análisis completo del exoma en trío de los progenitores no se detectó dicha variante (SYNLAB, Exámenes y valoraciones, 13 de julio de 2020. Los resultados se conservan en el Servicio de Pediatría del Hospital Universitario San José en Popayán, Colombia).

Casi todas las variantes patógenas de la secuencia de *PURA* reportadas hasta la fecha han sido *de novo,* el riesgo de que los hermanos la hereden parece ser poco (estimado empíricamente en mayor del 1 %), pero mayor que en la población general, debido a la posibilidad de mosaicismo de la línea germinal de los padres. Se han identificado muy pocos adultos con trastornos del desarrollo neurológico relacionado con *PURA* y ninguno ha tenido hijos. Sin embargo, el riesgo teórico para la descendencia de un individuo afectado es del 50 %, ya que esta enfermedad se hereda de forma autosómica dominante ^15^.

Las manifestaciones clínicas en el presente caso se iniciaron en el periodo neonatal y se caracterizaron por hipotonía acentuada de predominio axial, hipoactividad, movimientos distónicos, dificultad respiratoria con episodios de apneas y trastornos de la succión y la deglución.

En la mayoría de los reportes, se asegura que la hipotonía, la dificultad para la deglución y la apnea o hipoventilación primaria son los trastornos más comunes en el síndrome PURA, y los principales motivos por los cuales se hospitalizan estos pacientes ^11^^,^^15^. En una serie de 32 casos, se presentó hipotonía en el 97 %, dificultades para la deglución en el 81 % y dificultad para respirar en el 48 %. Se destaca el hecho de que la hipotonía es una de las manifestaciones más comunes en el síndrome PURA; por lo tanto, el abordaje del recién nacido hipotónico debe contemplar un amplio espectro de enfermedades posiblemente asociadas con trastornos genéticos ^19^.

Durante la edad escolar, se observó retraso en el desarrollo neurológico del paciente que se manifestaba con dificultades en el lenguaje. En la mayoría de los casos (alrededor del 91 %), los niños con el síndrome PURA sufren afasia, aunque tienen un buen lenguaje receptivo y pueden seguir instrucciones simples, pero presentan una falta notable de lenguaje expresivo. Otras manifestaciones que se deben tener en cuenta son epilepsia, estrabismo bilateral y un deterioro neurológico progresivo que eventualmente empeora el cuadro clínico ^8^^,^^20^.

## Conclusiones

La mayoría de las enfermedades huérfanas o raras son de origen genético. El avance y la implementación de los métodos de diagnóstico molecular han permitido que los pacientes afectados puedan tener un enfoque terapéutico más claro abren camino para continuar investigando estas enfermedades de tan baja prevalencia.

Gracias a métodos como la secuenciación completa del exoma, se han podido caracterizar pacientes con el síndrome PURA en todo el mundo y se han encontrado más variantes de secuencia patogénica *de novo.* Esto último ha permitido definir la importancia de aminoácidos muy conservados en la proteína alterada, lo cual ha establecido la importancia de las posiciones muy conservadas de los aminoácidos en la proteína afectada.

En el enfoque clínico de un paciente pediátrico hipotónico, y para la consideración de diagnósticos diferenciales, es importante elaborar una historia clínica completa con indagación de variables familiares, ambientales y demás, así como un oportuno estudio interdisciplinario. En este informe, el paciente presentaba síntomas asociados con haploinsuficiencia de la proteína PUR-a, causada por una variante heterocigota, de secuencia patogénica *de novo,* de tipo cambio de sentido. Mediante el enfoque adecuado de los hallazgos clínicos y el reporte molecular, se llegó al diagnóstico del paciente.

A nivel mundial, los sistemas de salud han puesto en marcha estrategias para que los escasos pacientes reportados con este síndrome mejoren su calidad de vida, ya que el tratamiento tiene un enfoque de manejo integral para evitar complicaciones causadas por las alteraciones de base descritas. En Colombia, es fundamental que esta enfermedad haga parte del listado de enfermedades huérfanas o raras del Ministerio de Salud y Protección Social.
